# Module-Based Analysis of Robustness Tradeoffs in the Heat Shock Response System

**DOI:** 10.1371/journal.pcbi.0020059

**Published:** 2006-07-28

**Authors:** Hiroyuki Kurata, Hana El-Samad, Rei Iwasaki, Hisao Ohtake, John C Doyle, Irina Grigorova, Carol A Gross, Mustafa Khammash

**Affiliations:** 1 Department of Bioscience and Bioinformatics, Kyushu Institute of Technology, Iizuka, Fukuoka, Japan; 2 Department of Biochemistry and Biophysics, University of California San Francisco, San Francisco, California, United States of America; 3 California Institute for Quantitative Biomedical Research, University of California San Francisco, San Francisco, California, United States of America; 4 Department of Biotechnology, Graduate School of Engineering, Osaka University, Osaka, Japan; 5 Control and Dynamical Systems, California Institute of Technology, Pasadena, California, United States of America; 6 Department of Cell and Tissue Biology, University of California San Francisco, San Francisco, California, United States of America; 7 Department of Microbiology and Immunology, University of California San Francisco, San Francisco, California, United States of America; 8 Department of Mechanical Engineering, University of California Santa Barbara, Santa Barbara, California, United States of America; Boston University, United States of America

## Abstract

Biological systems have evolved complex regulatory mechanisms, even in situations where much simpler designs seem to be sufficient for generating nominal functionality. Using module-based analysis coupled with rigorous mathematical comparisons, we propose that in analogy to control engineering architectures, the complexity of cellular systems and the presence of hierarchical modular structures can be attributed to the necessity of achieving robustness. We employ the Escherichia coli heat shock response system, a strongly conserved cellular mechanism, as an example to explore the design principles of such modular architectures. In the heat shock response system, the sigma-factor σ^32^ is a central regulator that integrates multiple feedforward and feedback modules. Each of these modules provides a different type of robustness with its inherent tradeoffs in terms of transient response and efficiency. We demonstrate how the overall architecture of the system balances such tradeoffs. An extensive mathematical exploration nevertheless points to the existence of an array of alternative strategies for the existing heat shock response that could exhibit similar behavior. We therefore deduce that the evolutionary constraints facing the system might have steered its architecture toward one of many robustly functional solutions.

## Synopsis

Biological systems maintain phenotypic stability in the face of various perturbations arising from environmental changes, stochastic fluctuations, and genetic variations. This robustness, which seems to be an inherent property of such systems, is still poorly understood at the molecular level. At the same time, systems approaches that were used with great success in the study and design of complex engineered systems provide a unique opportunity for investigating the basic tenants of robustness in cellular mechanisms. This is motivated by the fact that at the system level, biology and engineering seem to have a large number of common features despite their extremely different physical implementations. The heat shock response is one such robust cellular system, which interestingly achieves its seemingly simple objective of refolding or eliminating heat-denatured proteins through a complicated set of interactions. In analogy to engineering control architectures, the complex regulation strategies seem to be a specifically designed solution to generate robustness against different types of perturbations.

## Introduction

System-level approaches in biology have both a long history [1] and a revived present [2–6]. The main catalyst behind the renewed mainstream interest in the application of such approaches to the study of biological systems is their undeniable successes in the study of engineering systems. Indeed, systems-level design has consistently been at the core of modern engineering, motivating its most sophisticated theories in controls, computation, and information. While biological systems seem to employ feedback and feedforward loops of a similar nature to those present in engineering systems, they still possess subtle differences that need to be elucidated.

Whether applied to manmade or naturally occurring systems, the hallmark of a systems approach resides in the identification of distinct functional modules and protocols, i.e., the laws used to manage the connections between the different modules. In this context, two questions are of primary importance. What architectural aspects of biological modules make them similar or different from manmade machines [7,8]? And, how do biological control modules evolve and assemble into hierarchical modular systems that can survive in demanding environments [9]?

The first property that engineering and biological systems seem to have in common is the need for elaborate designs to generate robustly operational systems. A close scrutiny of both types of systems shows that in many instances, minimal designs are sufficient to generate nominal functionality. However, these minimal designs often fail to provide crucial aspects of robustness and performance necessary for competitive survival in challenging environments. Examples from technological sciences are abundant, while examples from natural sciences are still emerging [10–12]. In either case, a broad spectrum of strategies has evolved to generate such robustness and maintain internal conditions in the presence of both internal and external disturbances.

The heat shock response is one such robust system [13]. Heat shock causes unfolding, misfolding, or aggregation of proteins at the cellular level on the order of seconds, compromising cellular function. Cells overcome the heat stress by initiating the production of heat-shock proteins (hsps) that act as chaperones to refold denatured proteins into their native states, or proteases that degrade them. Although the objective of the heat shock response seems simple, its implementation involves complicated interactions. In *E. coli,* these interactions are centered on regulating the synthesis, degradation, and activity of the σ^32^ transcription factor [14]. These interactions result in chaperone-mediated and protease-mediated feedback loops, and a feedforward loop for translation [15]. Chaperone-mediated feedback measures the quantity of unfolded proteins and modulates the activity of σ^32^, whereas protease-mediated feedback modulates the stability of σ^32^. At the same time, the feedforward control senses temperature changes directly and instantaneously induces σ^32^ translation, thereby regulating σ^32^ synthesis.

Using a dynamic model of the heat shock response, we have previously analyzed the function of these feedback and feedforward regulators in terms of robustness [10]. The analysis presented in [10] mostly focused on local robustness properties corresponding to a particular choice of plausible parameter values. Here we explore instead a large space of relevant kinetic parameters and study the performance of the system in terms of newly defined mathematical criteria such as the efficiency and yield of the time response. These criteria are used to enable mathematical comparisons between the dynamics of wild-type and virtual knockout mutants that lack specific regulators. To interpret our results, we decompose the architecture of the system into hierarchical modules, and introduce the notion of a mechanistic protocol that dictates the way by which these modules are assembled.

## Results

### Modular Architecture in the Heat Shock Response System

**Figure 1:**
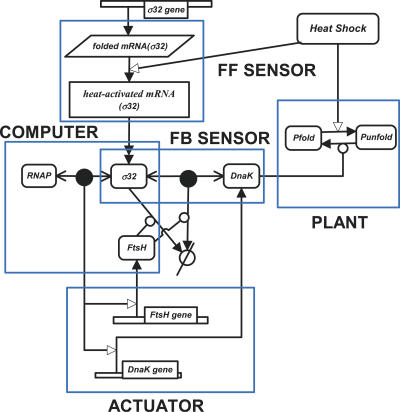
A Schematic Diagram of the E. coli Heat Shock Response and Its Functional Modules The notation of CADLIVE is used for simplifying the diagram: 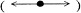 binding, 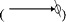 degradation, 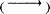 irreversible conversion, 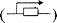 protein synthesis, 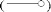 catalysis, 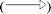 activation. The rectangles show the functional modules. The activation is the semantic reaction whose molecular mechanism is neither revealed nor fully described. For example, the notation of protein synthesis is semantic because many molecules, such as ribosomes, tRNAs, and amino acids, are omitted. On the other hand, the catalysis is the mechanistic reaction whose mechanism can be described at the molecular interaction level.

**Figure 2:**
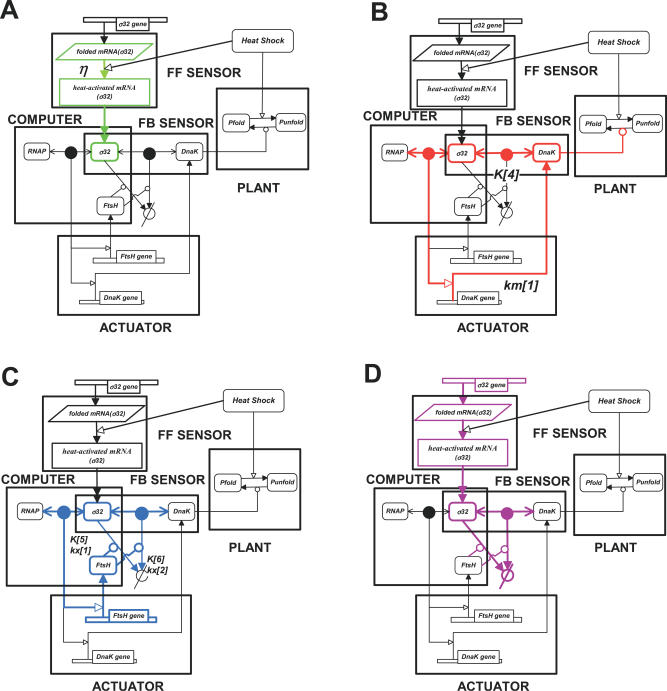
Flux Modules of the E. coli Heat Shock Response (A) Feedforward flux module. (B) Sequestration-mediated feedback (SEQ-FB) flux module. (C) Degradation-mediated feedback (DEG-FB) flux module. (D) σ^32^ amplifier flux module. The notation used is identical to Figure 1.

The biochemical map of the E. coli heat shock response can be decomposed into functional modules (Figure 1) and flux modules (Figure 2). The functional decomposition is carried out in analogy to engineering control systems block diagrams. Specifically, if we define the protein-folding task as the process to be regulated or controlled, then the quantity that drives this process is the actuation signal. The actuator module in the heat shock system comprises the high-gain processes of transcription and translation that produce cellular chaperones. Such synthesis of chaperones uses a modest control input, the number of free σ^32^, and amplifies it to produce a large chaperone actuation signal that drives the folding plant. This control signal is itself the output of a “computational” or a “controller” unit which, based on the sensed folding state of the cell, modulates the number of the σ^32^ molecules to generate an appropriate control signal. Finally, the folding state of the cell is sensed through the binding of the unfolded proteins to the chaperones. For example, when the number of unfolded proteins increases, the number of free chaperones decreases. The σ^32^ computational unit then assesses this measured signal. The information content of this signal leads, through both the degradation of the σ^32^ factor and the amount of sequestered σ^32^, to an adequate control action. The integration of the molecular modules into these defined functional modules is pictorially shown in Figure 1. The mathematical equations can also be connected to the functional modules for the full model (Tables 1–3, see also Materials and Methods). In this model, Equations 1–6 and 8–11 in Table 1 describe the computation module, while Equations 4 and 7 in Table 1 describe the FB sensor module. The mass balance equations, Equations 12–19 in Table 1, are common to both modules. The differential equations, Equations 20–28 in Table 1, describe the actuators and plants. To clearly illustrate such connections, a reduced order model of the heat shock response (Table S1) is divided into functional modules (Figure S1).

**Table 1:**
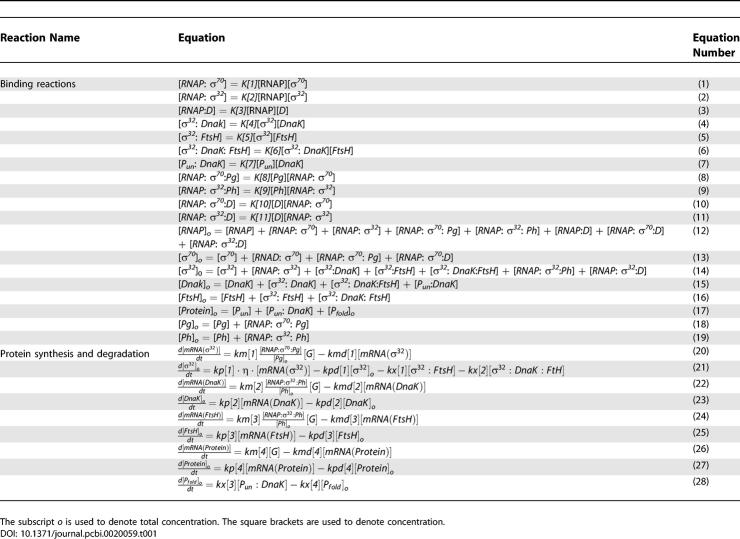
Mathematical Equations for the Detailed Mechanistic Model of the Heat Shock Response

Superimposed on these functional modules, we identify four distinct flux modules. Although our description of these fluxes will be mainly qualitative, the components of the fluxes can be easily identified in Figure 2 or through the equations describing the simplified heat shock model (Figure S2). It turns out that the identified roles of these fluxes are in close correspondence with different experimentally identified aspects of the regulation scheme of the heat shock response. First, we identify the FF flux module through which heat shock directly increases the translation rate for σ^32^ in a feedforward manner (Figure 2A). This flux affects the time response to a heat disturbance. Second, we identify the main feedback flux (SEQ-FB module), which acts through the interaction of σ^32^ with the chaperones (Figure 2B). The sequestration of σ^32^ is controlled by the folding state of the cell, and therefore the sequestration flux encompasses the folding machinery that dictates the number of free chaperones, and accordingly the number of free σ^32^ that are capable of binding to the RNA polymerase (RNAP) and initiating transcription of the hsps. Therefore, the function of this flux seems to be the regulation of the activity of σ^32^. The third flux module that we identify in the heat shock system is the degradation flux (DEG-FB module) (Figure 2C). The molecules involved in this flux are the free σ^32^ factors that, as a result of their sequestration by the chaperones, are susceptible to degradation by the FtsH protease. This flux essentially controls the stability of σ^32^. Finally, the fourth flux module is an “amplifier flux” (Figure 2D). The amplifier flux depicts the sequence of reactions that σ^32^ undergoes, in addition to its different encounters with other molecules from its synthesis to its degradation by FtsH. In the full model, the differential equations, Equations 20 and 21 in Table 1, describe the amplifier flux module, where the first term of Equation 21 describes the FF flux module. Equations 22 and 23 and Equations 24 and 25 (in Table 1) correspond to the SEQ-FB and DEG-FB flux modules, respectively.

### Speed, Yield, and Efficiency of the Heat Shock Response and Its In Silico Mutants

Having identified the modular structure of the heat shock response system and constructed the mathematical model, we now analyze the dynamic behavior of the three flux modules: SEQ-FB, DEG-FB, and FF. The main objective of the heat shock regulatory system is to refold denatured proteins upon exposure of the cells to heat shock. The response of the system is required to be fast, efficient, and robust. To capture these properties of the system, here we introduce the criteria for analysis: yield, efficiency, and response time (see Materials and Methods for details). Robustness and its connection to these criteria are analyzed in the next section. The yield is defined as the fraction of folded proteins in a pool of total proteins and the efficiency as the ratio of chaperones (DnaK) that are actively involved in refolding proteins to the total amount of chaperones. Both high efficiency and yield would indicate that unfolded proteins are being efficiently refolded with an appropriate number of hsps, a highly desirable feature. The speed of the response to increases in temperature also constitutes a crucial performance criterion in the heat shock system since proteins denatured for extended periods of time tend to form aggregates. We therefore define the response time as the time required for refolding 90% of the total folded proteins after heat shock and use it as a performance criterion.

To characterize the behavior of the system in terms of these performance criteria, we base our analysis on mathematical comparisons between wild-type and virtual mutants where various flux modules are disabled. The “virtual” mutants employed in this paper are distinct from ordinary gene knockout mutants. Genetic mutations alter the structure of the system while leaving all kinetic parameters not related to the mutation intact. Our virtual mathematical mutants, however, compensate for the mutation by readjusting the kinetic parameter values so as to conserve such properties of the wild-type as yield and efficiency. This allows for the direct comparison between architectures that generate an equivalent output, a difficult task in the wet lab. Mathematically, we carry out these mathematical mutations by looking at the structural blueprint of the heat shock response system and removing the terms in the differential equations model that contribute to the targeted mechanism. These virtual mutants include a system where FF is the only regulator (SEQ-FB and DEG-FB double knockout mutant), a system where sequestration is the only response regulator (FF and DEG-FB double knockout mutant), a system where sequestration is augmented with degradation feedback (FF knockout mutant), and a wild-type system possessing the three types of control. The kinetic parameters for the wild-type are determined from the heat shock literature. The kinetic parameters used in all mutant models are chosen to produce a value of the yield equal to 0.99 at both low and high temperature and an efficiency value greater than 0.9 at low temperature. We carry out the comparison between wild-type and mutants at high temperature. There are evidently multiple parameter sets that give a yield value equal to 0.99. We use these different sets to further study the dependence of the transient response on the kinetic parameters.

**Table 2:**
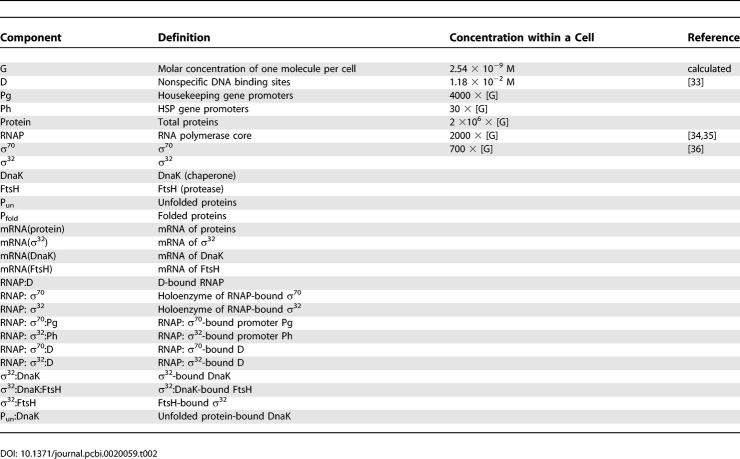
Components Used in the Detailed Mechanistic Model of the Heat Shock Response

**Table 3:**
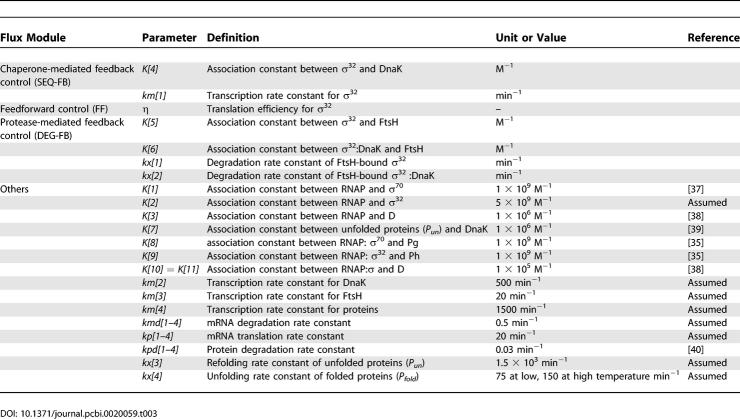
Kinetic Parameters Used in the Detailed Mechanistic Model of the Heat Shock Response

**Figure 3:**
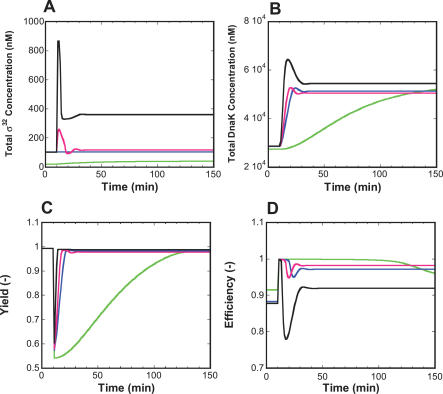
Time Evolution of σ^32^, DnaK, Yield, and Efficiency of the Heat Shock Response System and Its Different Mutants (A) σ^32^, (B) DnaK, (C) Yield, (D) Efficiency. Heat shock occurs at 10 min and is implemented through an increase in the rate constant for protein denaturing. The parameters for the wild-type and mutants are adjusted to provide a value of 0.99 for the yield at the steady state level at low temperature, and the efficiency is constrained to be more than 0.9 at high and low temperature. The values of the key parameters for each model are provided as follows. (Green) SEQ-FB/DEG-FB double mutant (FF system): *K[4]* = 0 M^−1^, *km[1]* = 0.04 min^−1^, *K[5]* = *K[6]* = 0 M^−1^, *kx[1]* = *kx[2]* = 0 min^−1^, *η* = 1 (low temperature), *η* = 4 (at high temperature); (Blue) DEG-FB/FF double mutant (SEQ-FB system): *K[4]* = 4.096 × 10^8^ M^−1^, *km[1]* = 0.2 min^−1^, *K[5]* = *K[6]* = 0 M^−1^, *kx[1]* = *kx[2]* = 0 min^−1^, *η* = 1; (Red) FF mutant (SEQ-FB + DEG-FB system): *K[4]* = 4.0 × 10^5^ M^−1^, *km[1]* = 1.6 min^−1^, *K[5]* = *K[6]* = 1.0 × 10^8^ M^−1^, *kx[1] = kx[2]* = 5 min^−1^, *η* = 1; (Black) Wild-type (SEQ-FB + DEG-FB + FF system): *K[4]* = 4.0 × 10^5^ M^−1^, *km[1]* = 1.6 min^−1^, *K[5]* = *K[6]* = 1.0 × 10^8^ M^−1^, *kx[1]* = *kx[2]* = 5 min^−1^, *η* = 1 (low temperature), *η* = 4 (at high temperature).

The corresponding time courses for wild-type and various mutants are shown in Figure 3. The level of σ^32^ for the wild-type in Figure 3A increases rapidly at heat shock, achieves a sharp peak, and then reduces to a new steady state. This behavior corresponds to a time course characteristic of a typical heat shock response. The FF knockout mutant model also produces a peak of σ^32^, albeit much smaller than in wild-type. This peak is due to the transient stabilization of σ^32^ by the DEG-FB module. In contrast, the system possessing only FF (the SEQ-FB/DEG-FB knockout double mutant) exhibits an over-damped response and does not show any peaks in the level of σ^32^ upon heat shock. The slow rise in σ^32^ is due to increased translation at high temperature. When the only form of regulation is through the SEQ-FB (the DEG-FB/FF double knockout mutant), the level of σ^32^ decreases slightly after heat shock as the abundant free σ^32^ at the onset of heat shock competes with the housekeeping σ^70^ factor to bind RNAP. As a result, the production of σ^32^, which is governed by σ^70^, is slightly suppressed. It is therefore apparent that DEG-FB contributes greatly to the characteristic peak of σ^32^. Our model does not take into account the fact that σ^32^ is also transcribed by the sigma factor σ^E^. This transcription is negligible at 30 °C and increases at 42 °C. Further, transcription of σ^70^ increases after heat shock since σ^70^ has a heat shock promoter. Overall, this contributed in part to the experimentally observed increase in σ^32^ levels upon heat shock induction. However, as a first approximation, we expect the contribution of these interactions to be small compared with the contribution of the DEG-FB.

Figure 3B shows the time course for the chaperone DnaK. The level of DnaK for the wild-type system increases upon heat shock, achieves a small peak, and then declines to a new steady state. The DnaK time courses for the FF mutant and the DEG-FB/FF double mutant show smaller peaks and delayed rise times, while that for the SEQ-FB/DEG-FB double mutant shows a critically delayed response.

Figure 3C shows the yield time course that characterizes the transient response of folded proteins after heat shock. The figure indicates that the level of folded proteins in all four systems was satisfactorily restored to its pre-shock value at the end of the response. The response in the FF mutant and DEG-FB/FF double mutant was slightly delayed, while the delay in the SEQ-FB/DEG-FB double mutant was about 100 min. These results show that FF alone is not sufficient to achieve the fast response observed in the wild-type heat shock. Alternatively, the FF, SEQ-FB, and DEG-FB modules orchestrate their functions and work additively to implement a fast response.

Figure 3D shows the efficiency time course that characterizes the transient response of folded proteins after heat shock. Upon temperature up-shift, the efficiency increases sharply as heat-denatured proteins accumulate and bind to DnaK. In all the cases considered, except for the SEQ-FB/DEG-FB double mutant, the efficiency undergoes an under-damped transient and finally reaches a new steady state. The shape of the transient is negatively correlated with that of the DnaK concentration, as expected from the definition of the efficiency measure. As the DnaK level overshoots slightly, an excess of DnaK is produced, generating disproportionate increases in the total number of DnaK molecules as compared with the DnaK level bound to unfolded proteins, therefore reducing the efficiency. Comparison of the yield and efficiency plots points to a fundamental tradeoff that underlies the operation of the heat shock response system. While overexpression of DnaK increases the yield, it simultaneously decreases the efficiency, which necessitates the existence of a balance that establishes acceptable values for both.

**Figure 4:**
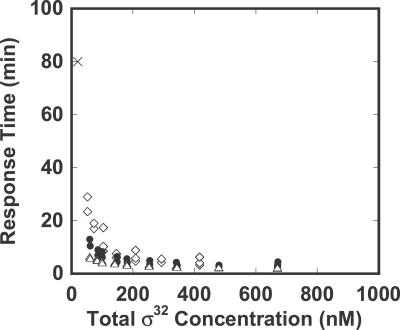
Transient Response of the Wild-Type and Virtual Mutants The response time, defined as the time at which the level of unfolded proteins recovers to within 90% of its pre-shock value, is plotted versus the total σ^32^ concentration at low temperature. The level of σ^32^ parameterizes the 2-D parameter space (the transcription rate constant for σ^32^ and the association constant between σ^32^ and DnaK) that satisfies the yield and efficiency constraints (yield > 0.9 and efficiency > 0.85 at low and high temperature). The two parameters:

We now quantify more accurately some of our previous observations. Specifically, we focus on the FF and DEG-FB loops, since Figure 3CD indicates that they greatly influence the adaptation time of the yield and efficiency. For the DEG-FB/FF double mutant, the FF mutant, the SEQ-FB/DEG-FB double mutant, and the wild-type models, we compute both the response time and low temperature σ^32^ concentration for values of the 2-D parameter space (corresponding to the transcription rate constant of σ^32^ and the association constant between DnaK and σ^32^) that achieve a yield greater than 0.9 and an efficiency greater than 0.85. The result is shown in Figure 4. For the SEQ-FB/DEG-FB double mutant (FF-alone system), the response time was 80 min. It was difficult to obtain a fast response under the provided constraints of the yield and efficiency. However, the addition of FF to the closed loop feedback system substantially reduced the response time. For the values of the parameters that achieve low σ^32^ concentrations, the addition of DEG-FB to SEQ-FB dramatically reduced the response time, an effect further enhanced by the addition of FF. However, the response time in the three cases was comparable at high σ^32^ concentrations. SEQ-FB with the high σ^32^ concentration showed a fast response, because the heat shock response does not need the time-consuming synthesis of σ^32^.

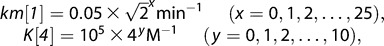
were varied for the wild-type (SEQ-FB + DEG-FB + FF) (▵): *K[5]* = *K[6]* = 1 × 10^8^ M^−1^, *kx[1]* = *kx[2]* = 5 min^−1^, *η* = 1 (low), *η* = 2 (high), for the DEG-FB/FF double mutant (SEQ-FB) (⋄): *K[5]* = *K[6]* = 0 M^−1^, *kx[1]* = *kx[2]* = 0 min^−1^, *η* = 1, and for FF mutant (SEQ-FB + DEG-FB) (•): *K[5]* = *K[6]* = 1 × 10^8^ M^−1^, *kx[1]* = *kx[2]* = 5 min^−1^, *η* = 1. For SEQ-FB/DEG-FB double mutant (FF alone) (X): *K[4]* = *K[5]* = *K[6]* = 0 M^−1^, *kx[1]* = *kx[2]* = 0 min^−1^, *η* = 1 (low), the two parameters of *km[1]* and *η* (high temperature) are varied as:

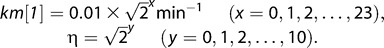
In the mutant where FF is operating alone in open loop, the parameter combination of *km[1]* = 0.04 min^−1^ and *η* = 1 (low), *η* = 2 (high) is the only solution to satisfy the required yield and efficiency.

### Sensitivity Analysis

In this section, we study the robustness properties of the heat shock response. Specifically, we focus on the sensitivity of the total chaperone level to the transcription rate constant of DnaK. A large sensitivity of the number of chaperones to crucial parameters is undesirable as it produces a variable and unpredictable folding of proteins, both at low and high temperature. We again compare the various mutants and the wild-type. For the SEQ-FB/DEG-FB double mutant, we search the 2-D parameter space corresponding to the translational efficiency of σ^32^ (parameter *η* in Table 3) and the transcription rate constant of σ^32^ (parameter *km[1]* in Table 3). We isolate the values of these parameters that produce acceptable values of yield and efficiency at low and high temperature (set again to values greater than 0.9 and 0.85, respectively). For such values of the parameters, we determine the sensitivity of DnaK to its transcription rate (parameter *km[2]* in Table 3). Interestingly, the SEQ-FB/DEG-FB double mutant exhibited only one set of parameter values that were able to reproduce the desired yield and efficiency. Obviously, the sensitivity of the DnaK level to its transcription rate in the SEQ-FB/DEG-FB double mutant is one since all parametric perturbations are directly transmitted to the output of the system in the absence of feedback loops. However, in addition to large sensitivity, these results indicate that the absence of feedback limits the kinetic range where the system can operate efficiently.

**Figure 5:**
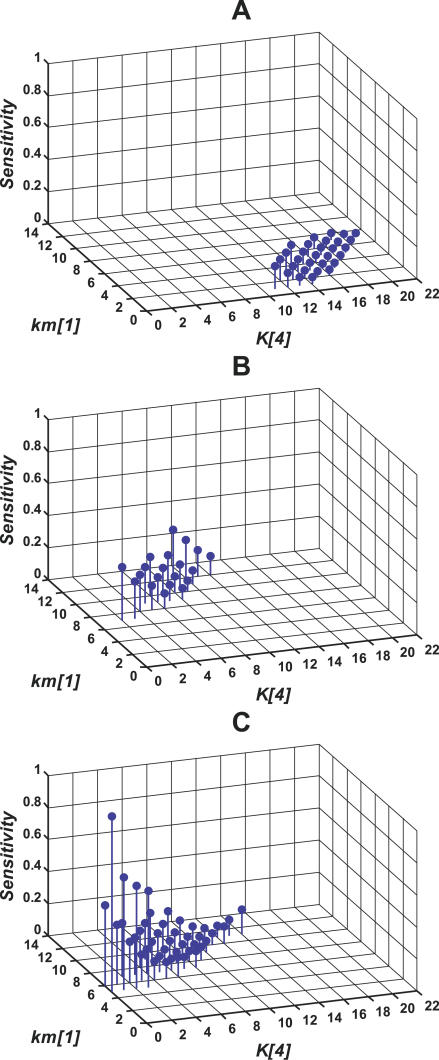
Sensitivity of the Total DnaK to the Change in the Transcription Rate Constant for DnaK at Low Temperature The plot reports the sensitivity computed for the parameters values that satisfy the required yield and efficiency (yield > 0.9 and efficiency > 0.85 at low and high temperature). Yield and efficiency were computed by searching the 2-D parameter space consisting of the transcription rate constant for σ^32^ and the association constant between σ^32^ and DnaK as follows:

We repeat the same exercise for the other virtual mutants. For the various cases considered, we search the 2-D parameter space corresponding to the binding between σ^32^ and DnaK (*K[4]* in Table 3) and transcription rate of σ^32^ (*km[1]* in Table 3), and isolate regions that simultaneously produce values of yield greater than 0.9 and values of efficiency greater than 0.85 at both low and high temperatures. For these parameter regions, we determine the sensitivity of DnaK to its transcription rate during low-temperature growth. The FF control operating alone in open loop is characterized by a high sensitivity of the total DnaK to the transcription rate constant for DnaK (unpublished data). This result is expected, as lack of robustness is an inherent property of open loop systems. By contrast, a system implementing SEQ-FB is characterized by small sensitivity, demonstrating that SEQ-FB plays a major role in enhancing robustness (Figure 5A). Interestingly, the addition of DEG-FB to SEQ-FB shifts the parameter region where the desired yield and efficiency can be achieved (Figure 5AB). In fact, the increased synthesis of FtsH at high temperature suppresses the level of σ^32^, resulting in decreased yield as compared with the mutant where SEQ-FB is the only control mechanism available. For parameters lying in the desired yield and efficiency region, the sensitivity is slightly higher than in the system implementing SEQ-FB alone. The addition of the FF loop to DEG-FB and SEQ-FB increases the size of the parameter space where yield and efficiency can be achieved, but does not contribute to the decreased sensitivity (Figure 5BC). This effect is the outcome of the ability of FF to generate high values for the yield at elevated temperatures. The sensitivities in these three cases did not significantly depend on the σ^32^ concentration (unpublished data), differing from the case of transient response.

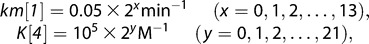
for the various systems, (A) DEG-FB/FF double mutant (SEQ-FB system): *K[5]* = *K[6]* = 0 M^−1^, *kx[1]* = *kx[2]* = 0 min^−1^, *η* = 1, (B) FF mutant (SEQ-FB + DEG-FB system): *K[5]* = *K[6]* = 1 × 10^8^ M^−1^, *kx[1]* = *kx[2]* = 5 min^−1^, *η* = 1, (C) Wild-type (SEQ-FB +DEG-FB + FF system): *K[5]* = *K[6]* = 1 × 10^8^ M^−1^, *kx[1]* = *kx[2]* = 5 min^−1^, *η* = 1 (low), *η* = 2 (high).

**Figure 6:**
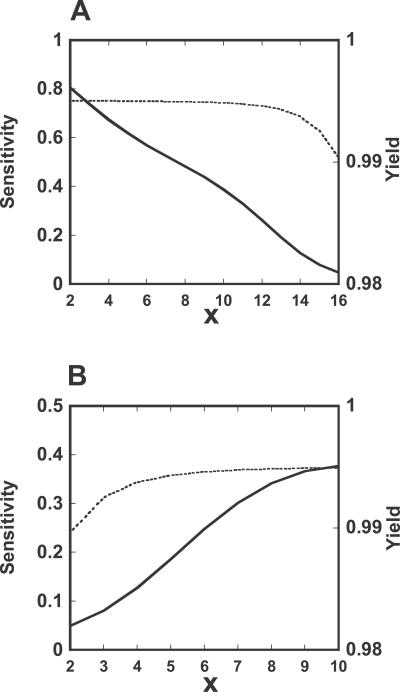
Robustness Tradeoff of SEQ-FB The plots show the yield (dotted line) and the sensitivity of the total DnaK concentration (solid line) with respect to a change in the transcription rate constant for DnaK as a function of the association between and σ^32^ and DnaK (*K[4]*) (A) and the transcription rate constant for σ^32^ (*km[1]*) (B). (A) The association constant between σ^32^ and DnaK (*K[4]*) is varied as follows, *K[4]* = 10^5^ × 2*^x^* M^−1^ (*x* = 2, …, 16), when the other kinetic parameters are fixed: *km[1]* = 0.8 min^−1^, *K[5]* = *K[6]* = 0 M^−1^, *kx[1]* = *kx[2]* = 0 min^−1^, and *η* = 1. (B) The transcription rate constant for σ^32^ (*km[1]*) is varied as follows, *km[1]* = 0.05 × 2*^x^* min^−1^ (*x* = 2, …,10), when the other kinetic parameters are fixed: *K[4]* = 1.6 × 10^9^ M^−1^, *K[5]* = *K[6]* = 0 M^−1^, *kx[1]* = *kx[2]* = 0 min^−1^, and *η* = 1.

To further delineate this tradeoff between yield and sensitivity, we computed the sensitivity of the total DnaK and the yield of the response to *K[4],* the association constant between σ^32^ and DnaK (Figure 6A), and to *km[1],* the transcription rate constant for σ^32^ (Figure 6B). An increase in *K[4]* decreases both the sensitivity and the yield, while an increase in *km[1]* increases both. However, while the sensitivity value changes by multiple folds over the range of *K[4]* and *km[1]* variation, the changes in the yield values are more modest. Therefore, an increase in the “strength” of the SEQ-FB greatly increases the robustness of the system, but at the expense of some decrease in its yield performance. This can be analytically validated further using the simplified heat shock model (Figure S3, Protocol S1).

### Stochastic Analysis

**Figure 7:**
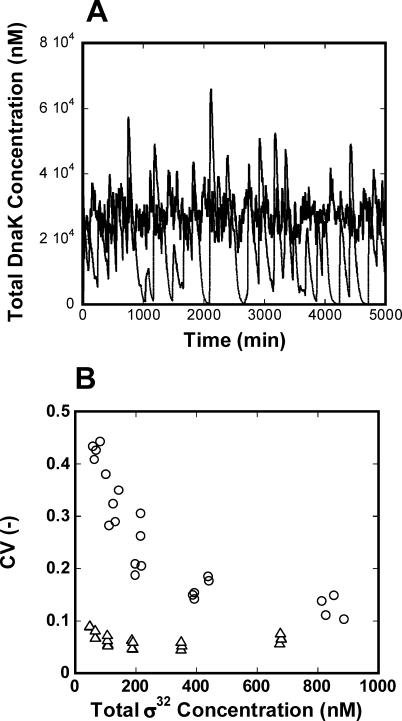
Stochastic Simulation of the Heat Shock Response (A) Stochastic realization of the concentration of DnaK at low temperature for the wild-type and the DEG-FB mutant. The average concentration of σ^32^ and DnaK are constrained to be equal in wild-type and mutant. In the wild-type (bold line): *K[4]* = 5.12 × 10^7^ M^−1^, *km[1]* = 3.2 min^−1^ *K[5] = K[6]* = 1 × 10^8^ M^−1^, *kx[1] = kx[2]* = 5 min^−1^, η = 1. In the DEG-FB mutant (thin line): *K[4]* = 5.12 × 10^7^ M^−1^, *km[1]* = 0.1 min^−1^ *K[5] = K[6]* = 0 M^−1^, *kx[1] = kx[2]* = 0 min^−1^, η = 1. (B) The CV of DnaK is plotted as a function of total σ^32^ concentration for the wild-type (SEQ+DEG+FF system) (▵) and the DEG-FB knockout mutant (SEQ+FF) (○). The plot reports the CV for the parameter values that satisfy the required yield (>0.9) and efficiency (>0.85). Yield and efficiency are computed by searching the 2-D parameter space consisting of the transcription rate constant for σ^32^ and the association constant between σ^32^ and DnaK as follows,

The effect of stochastic fluctuations attributed to biochemical noise has been shown to be especially pronounced in systems with low molecular counts [16]. The main regulator of the heat shock response, σ^32^, is on the order of 30–50 molecules per cell (75–125 nM). We have previously demonstrated that DEG-FB contributes largely to the attenuation of biochemical noise generated by the small numbers of σ^32^ molecules [10,17] (see also Figure 7A, computed using the Stochastic Simulation Algorithm [18]). Here, we extend this analysis by comparing the stochastic behavior of the wild-type heat shock system implementing DEG-FB to that of a DEG-FB mutant for a large range of parameters. As a measure of the variability in the system, we consider the coefficient of variation (CV) of DnaK, calculated by dividing the standard deviation of DnaK time course data by its mean value. Similar to the deterministic analysis, we only consider the parameter values in the 2-D parameter space *(K[4]* and *km[1])* that achieve a yield value greater than 0.9 and an efficiency value greater than 0.85 at low and high temperatures. For the parameter values that achieve such desired efficiency and yield values, we compute the CV and the mean concentration of total σ^32^ at low temperature, and plot one versus the other. The result is shown in Figure 7B. For the wild-type, the CV is less than 0.1 over the whole range of σ^32^. In the DEG-FB knockout mutant, however, the CV is considerably larger, particularly for lower σ^32^ concentrations. At higher concentrations of σ^32^, the CV for both wild-type and mutant decreases are as would be expected. The CV of the wild-type remains smaller than that of DEG-FB mutant for all ranges of σ^32^ concentrations. The two CVs however become comparable for large concentrations of σ^32^, indicating that an alternative heat shock response strategy lacking DEG-FB can achieve reasonable noise attenuation by using a large number of σ^32^ molecules. Such a strategy would evidently entail a tradeoff in terms of the metabolic cost of producing numerous σ^32^, and hence numerous chaperones. Interestingly, the periplasmic heat shock response in E. coli is centered on the abundant sigma-factor σ^E^ (5,000 molecules/cell) whose degradation is not regulated [19].

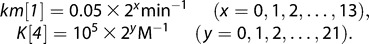

## Discussion

### Functional Analysis of the Heat Shock Response and Comparison with Engineering Systems

In this paper, we have used engineering analogies to decompose the heat shock system into hierarchical molecular, functional, and flux modules. Following this modular architecture, we found that mathematical mutations closely corresponded to the deletion of specific flux modules. The mathematical comparison between wild-type and the virtual mutants that lack flux modules proved to be very useful for assigning a function to the deleted components. To perform unbiased comparisons between wild-type and mutants, the mathematical models were constrained by the requirement of achieving the same values of appropriately defined yield and efficiency measures. Using a combination of analytical results, careful simulations, and searches in the relevant parameter space, we investigated the functions of each module in terms of performance criteria such as transient response, steady-state sensitivity and noise rejection characteristics. FF was identified as a powerful strategy that allows for the adaptation to elevated temperatures but that cannot implement a robust response if used by itself in open loop. Negative feedback loops increased the robustness of the system in the presence of parametric uncertainty and internal fluctuations, but limited the yield for production of hsps and hence the folding of heat-denatured proteins. Furthermore, the use of degradation feedback implemented a faster response to a heat disturbance and reduced the effects of biochemical noise [10,17].

Despite extremely different physical implementations, these regulatory strategies are widely used both in engineering and in biological systems. The electronic amplifier is an example of an engineering system that utilizes feedback to create robustness to parameter variations. In the absence of feedback, an open-loop amplifier has a wildly varying gain due to its sensitive dependence on its parameters and external environment. By closing the loop with the appropriate feedback, the system attains robustness to various uncertainties, making it a reliable amplification device that is used across a wide range of engineering applications. One often finds more than one layer of feedback control in engineering systems. For example, a feedback amplifier may itself be used in a feedback loop to control the motion of a mechanical device such as a motor. Feedforward control is also frequently employed in engineering systems to speed up the systems rejection of external disturbances. Once measured, these disturbances can be compensated for directly at the system input before they lead to large deviations in the output. Even though such deviations eventually get sensed and corrected by the feedback loop, the response time and effort needed for correction will be larger in the absence of feedforward. One engineering domain where feedforward is in common use is process control. As an example, a stirred tank with a heat exchanger is often used to maintain the reactants within the tank at a desired temperature. A feedback loop that adjusts the heat flow rate based on temperature measurement in the tank can be used for that purpose. If, in addition, the highly variable temperature of the feed material entering into the tank (disturbance) is also measured, this signal can be used in a feedforward fashion to adjust the heat flow rate in a way that cancels, or almost cancels, its anticipated effect on the tank temperature. Any residual differences between the actual and desired temperature is sensed by the feedback sensors and is corrected via the feedback loop. The result is a faster response and improved performance over feedback control alone.

As a result of this analysis, we propose that the use of complex regulation strategies in the heat shock system is likely to be a specifically designed solution to different aspects and requirements of heat remediation rather than the result of evolutionary accidents that gave birth to redundant regulatory loops. To the contrary, the regulatory structures in the heat shock response seem to be crucial elements whose function is orchestrated to address the numerous, and sometimes contradictory, performance and design issues. These regulatory structures in turn impose new tradeoffs and costs that can be identified in the heat shock system. The SEQ-FB, for example, causes the yield of the response to decrease. Furthermore, the DEG-FB module implements a futile synthesis/degradation cycle that generates a fast response and high feedback gain despite the expense of constant utilization of mass and energy. At the same time, the use of complex regulation strategies generates new fragilities that are then compensated by other modules. For example, an FtsH knockout causes σ^32^ to increase explosively in our model; DnaK would be overproduced to dangerous levels if there were no SEQ-FB module. Redundant protease flux modules (HslVU, ClpAP, Lon) appear to be fine-tuning for the level of σ^32^, but they do not fully compensate for mutations in FtsH since FtsH null mutants have very high levels of σ^32^ and a half life of 40–60 min [20,21]. Such tradeoffs between robustness and fragility seem to be a recurring theme both in biological systems and in the design of manmade machines.

### Alternative Strategies for the Implementation of the Heat Shock Response

The existing architecture of the heat shock response is not the unique solution to achieve robustness and fast response in a system whose objective is to rescue the cell upon protein unfolding in a timely manner. Indeed, our mathematical analysis indicates that a system implementing SEQ-FB with FF (with no DEG-FB) is able to reproduce these characteristics, albeit at the expense of a much higher level of σ^32^ concentration. Indeed, in *Proteus mirabilis,* a γ-proteobacteria closely related to *E. coli,* control of σ^32^ is implemented by SEQ-FB with FF but no DEG-FB. Importantly, as we predict, in this organism the levels of σ^32^ are high at 30 °C and change very little if at all upon shift to high temperature [22]. Likewise, only the activity of the E. coli abundant σ-factor (σ^E^) that implements the periplasmic heat shock response is regulated through its sequestration by the trans-membrane protein RseA [23]. These variations raise questions about how the DEG-FB strategy might have evolved, and the evolutionary constraints that led to its existence in the cytoplasmic heat shock response.

### Connections of Modularity and Protocols to Evolvability

Engineering sciences constantly exploit the properties of modular designs to rapidly advance technology. The basic mechanical or electrical strategies that form the functional core of a plane, a car, or a computer change only in rare instances. It is often the superimposition of new modules and their correct interface with older modules through standardized protocols that generate increased speed, reliability, safety, and robustness. Modularity guarantees that the complexity of a design is hidden in “black boxes” that possess well-defined inputs, outputs, and functionality. At the same time, standardized interfaces guarantee the plug-and-play addition of other modules, without the need for extensive fine adjustment to achieve coordination with the existing modules. Therefore, modularity and protocols are often prerequisites for smooth evolvability, and as such may also have been extensively used in the evolution of complex gene regulatory networks. For example, in the heat shock response system, σ^32^ acts as a hub that integrates various flux modules (FF, SEQ-FB, DEG-FB) in interconnected loops (Figure S4). Such architecture leaves room for evolvability in a simple manner. Imagine for instance that at some point in the evolutionary past of the heat shock system, FtsH synthesis was not controlled by σ^32^. Such a system, where the degradation of σ^32^ is not negatively regulated by a member of the σ^32^ regulon, would have experienced large stochastic variability, necessitating the evolution of a mechanism for noise attenuation. One possible scheme would have been to evolve an independent controller that stabilizes the levels of FtsH tightly around its desired value. Perhaps a more straightforward solution to the problem is to employ an interconnected loop whereby the FtsH module is evolved just by fusing a promoter for binding of σ^32^ to the FtsH gene [24,25] (Figure S5). Other proteases such as HslVU, ClpAP, and Lon are linked similarly to the σ^32^ molecular module, although these proteases seem to play little or no role in vivo in the degradation of σ^32^ [21]. Overall, approaches linking the architectural features of cellular networks, their functionality and performance, and their evolvability properties will undoubtedly be crucial for the investigation of biological complexity.

## Materials and Methods

### Molecular architecture in the heat shock response.

In fast-growing E. coli at 37 °C, the major sigma factor, σ^70^, binds RNAP core enzyme and directs RNAP to transcribe the genes necessary for normal growth. Heat shock (i.e., the increase in temperature to 42 °C) causes the amount of another sigma factor, σ^32^, to increase [26]. This in turn results in increased expression of the σ^32^-regulated hsps (chaperones and proteases). The heat shock response aims at refolding heat-denatured proteins through the action of chaperones or degrading such proteins by proteases as a measure to prevent them from forming aggregates. The heat shock response depends primarily on the regulation of σ^32^ activity, stability, and synthesis [13–15]. The activity of σ^32^ is regulated through its sequestration by chaperones. Chaperone-bound σ^32^ is prevented from binding to RNAP, which limits its transcription activity [26,27]. The stability of σ^32^ is regulated through its degradation by various proteases, predominantly FtsH, although HslVU, ClpAP, and Lon may play minor roles in degrading σ^32^ in vivo [19,20]. FtsH degrades σ^32^ in an ATP-dependent manner. The synthesis of σ^32^ is regulated at the translational level. The *rpoH* mRNA forms a stable secondary structure which prevents the initiation of translation at low temperature. Higher temperature disrupts this secondary structure, inducing translation [15]. Figure 1 depicts the molecular mechanisms involved in the heat shock response as described above. The detailed notation of biochemical reactions is defined elsewhere [28,29].

These interactions result in a time course of the heat shock response that proceeds as follows. Upon heat shock, there is an increase in the cellular levels of unfolded proteins that sequester the members of the chaperone team. This results in an increase in σ^32^ activity and stability. At the same time, the synthesis of σ^32^ increases, resulting in its accumulation and leading to hsp induction. Therefore, the level of σ^32^ rapidly increases, reaching a peak. Afterward, the level of active σ^32^ is reduced by chaperone-mediated sequestration and protease-mediated degradation of σ^32^ until it reaches a new steady state at high temperature.

### Detailed mechanistic model of the heat shock response.

Based on the interactions described above, we built a detailed mathematical model of the heat shock response (Table 1). The components and kinetic parameters of the model are shown in Table 2 and Table 3, respectively. In the model, we account for the regulation of σ^32^ activity through the chaperone-mediated feedback control (SEQ-FB), and for the regulation of σ^32^ through the protease-mediated feedback control (DEG-FB). We also include the feedforward control that regulates the efficiency of σ^32^translation as a step function of temperature (FF). We take DnaK as the representative of cellular chaperones and FtsH as representative of cellular proteases. There is data indicating that the GroE/GroL/GroS chaperone machinery is also involved in the inactivation of σ^32^. However, the details of this interaction are still an area of active research [30].

The model uses first order mass action kinetics to describe the synthesis, proteolysis, and binding of proteins. We make the common assumption that binding reactions occur on a faster timescale than production and degradation of proteins. Therefore, we replace the differential equations describing these fast reactions by algebraic binding equations. This mathematical model has been automatically built by using the CADLIVE system [31]. The kinetic parameters for the wild-type heat shock were picked or estimated from the vast heat shock literature. The simulated time course trajectories for σ^32^ and chaperone reproduced the qualitative behaviors of wild-type and mutants (Figure S6).

### Modular decompositions through modeling.

A module has been characterized as a subsystem that possesses a function that is separable from that of other modules, in the sense that it is capable of maintaining most of its identity when isolated or rearranged [4], or is defined as “groups of nodes [in a graph] that are relatively isolated from the rest of the system” [9]. We prefer an approach to modularity that emphasizes connectivity and function over isolation. Indeed, regulatory modules are not necessarily isolated, nor would they preserve their function if isolated or rearranged, except in very structured and organized ways [32]. To provide a useful characterization of modularity, it is imperative to classify and identify different types of modularity based on the level of detail or abstraction that one is adopting. The resulting multi-resolution scheme can then be used to assess different aspects of the modular decomposition, zooming out from the molecular description (molecular modules) to a block diagram–like picture (functional modules).

### Molecular modules: Zooming in to the fine details.

Molecular modules are defined as molecular entities that implement the mechanistic biological functionality and have identifiable interfaces and interactions with other molecular modules. For example, σ^32^ is an integral molecular module that integrates key regulation strategies. This definition of molecular modules is obvious in the sense that they are the entities investigated in experimental settings. The behavior of molecular modules is assumed to be stochastic in nature but their orchestrated operation is often observed to be highly robust and reliable.

### Functional modules: Zooming out to the block diagram.

At a lower level of resolution, the components of a system can be divided into “functional groups” that we refer to as *functional modules*. This functional decomposition is a routine procedure in disciplines like control engineering, where the systems considered often have levels of complexity almost comparable to biology (power networks, planes, industrial processes) [7]. Therefore, we seek analogies with the modules that are traditionally identified in control engineering schemes. In such systems, the process to be controlled or regulated is identified, and the rest of the network is classified in terms of the function that it accomplishes to facilitate this regulation. Following engineering terminology, we call the process to be regulated or controlled the “plant.” The other typical functional modules usually present in engineering systems include sensing and actuation modules, in addition to a “controller” module that actively computes the control signal based on the information provided by the other modules. The output of the plant to be controlled is usually sensed and the measurement provided to a logical unit, the controller. Based on this output measurement, the controller devises a control law, which is then fed to an actuator that drives the plant, therefore regulating its output. A block represents each of these modules, and the interconnection of such blocks is frequently referred to as a *block diagram*.

### Flux modules: Connecting the fine to the coarse.

A pathway is commonly defined as a linked set of biological reactions. The concept of a pathway is very useful in comprehending the networks involved in regulating the cell's vital functions. We define a flux module as a pathway that traces the fate and mechanisms of interaction of a group of molecules involved together in the performance of a certain function. A flux module ideally connects different functional modules, but forms an entity that possesses its own functionality. Therefore, a flux is essentially the flow of information in the network, accompanied by a function tag that characterizes this flow. Notice that different fluxes may use some parts of the same pathway to accomplish distinct and identifiable functions. In terms of the deterministic rate equations,
fluxes can be traced to be a collection of *f_ij_* that establish a flow of information with a specific functional role as defined above. Feedback loops are simple examples of flux modules.

### Criteria for system analysis.

The main objective of the heat shock regulatory system is to refold denatured proteins upon exposure of the cells to higher than normal temperatures. However, a response that accomplishes this function while, for instance, using an excess of hsps cannot be evolutionarily favorable, as the production and maintenance of these hsps represent an important metabolic burden. Therefore, cells must achieve a fine balance between the protective effect of the hsps and the metabolic burden of overexpressing them. At the same time, the response should be of appropriate speed and magnitude so as not to expose the cell to prolonged periods of damage, and robust in the presence of environmental changes, intrinsic noise, and intracellular interference from other cellular subsystems. For example, sigma factors including σ^32^, σ^38^, σ^54^, and σ^70^compete for binding to the limited number of RNAP [32]. The consequence of this competition for the heat shock response is that σ^32^ is constantly exposed to disturbances from other sigma factors.

We quantify these various design requirements and assess their implementation strategies by devising mathematical mutants where certain structures present in the wild-type heat shock are absent, then comparing the performance of the mutants to that of wild-type. The performance criteria used in this procedure are as follows.

### Comparison criteria and search for critical parameter space.

Since the objective of the heat shock response is to reduce the amounts of unfolded proteins, we define the yield of the response as the fraction of folded proteins (*P_fold_*) in a pool of total proteins (*P_total_*):



Although an excess amount of chaperone can be sufficient to refold proteins, hence increasing the yield, it can cause a wasteful synthesis burden for cell metabolism. Therefore, we define the efficiency of the response as the ratio of chaperones that are actively involved in refolding proteins (quantified by the amount bound to unfolded proteins *P_un_:DnaK*) to the total amount of chaperones in the cell:



Robustness in engineering design and analysis is measured by the system's ability to resume successful operation in the presence of signal and system uncertainties. We quantify the heat shock system's robustness to parametric fluctuations by calculating the sensitivity in the level of the chaperones to perturbations related to σ^32^. We define sensitivity by the steady-state logarithmic gain:
This sensitivity measure captures the amount of change in the total DnaK from *Y*_1_ to *Y*_2_ induced by a change in the transcription rate constant from *X*_1_ to *X*_2_.

The yield, efficiency, robustness, and speed of response criteria described above are used systematically to characterize the functions of the various structural components in the heat shock system and to constrain parameter values in wild-type and mutant heat shock models. For example, to characterize the transient response and the sensitivity at steady state, the yield and efficiency are constrained to be above a certain acceptable value. Comparison between wild-type and mutants is only made for values of the parameters in these models that yield a response satisfying the required yield and efficiency. These parameters values are chosen by gridding the appropriate parameter spaces over a wide range of values and computing the corresponding control performance criteria. In this analysis, we focus on the parameters of critical importance to the synthesis, sequestration, and degradation of σ^32^. For SEQ-FB, the transcription rate constant for σ^32^ (*km[1]*) and the association constant between σ^32^ and DnaK (*K[4]*) are selected as critical factors. For DEG-FB, the FtsH-mediated degradation rate constant for σ^32^ (*kx[1] = kx[2]*), the transcription rate constant for σ^32^ (*km[1]*), and the association constant between FtsH and σ^32^ (*K[5] = K[6]*) are employed. For FF, the critical factor is considered to be the translation efficiency (*η)*.

## Supporting Information

Figure S1 Mathematical Functional Decomposition of the Reduced Order Heat Shock System (75 KB PDF)

Figure S2 Connection of Flux Modules to the Differential Equations Describing the Reduced Qualitative Model (61 KB PDF)

Figure S3 Time Evolution of σ^32^ (*S_t_*) for a Reduced Qualitative Model of the Heat Shock Response (83 KB PDF)

Figure S4 Definition of Interconnected Feedback Loops (65 KB PDF)

Figure S5 Protocol for Interconnected Feedback Loops Evolution of the DEG-FB loop is used an example.
(70 KB PDF)

Figure S6 Time Evolution of σ^32^ and DnaK for a Detailed Mechanistic Model in Wild-Type and Mutants (70 KB PDF)

Protocol S1 Derivation of the Sensitivity Equation from the Reduced Qualitative Model (60 KB DOC)

Table S1 Reduced Qualitative Model of the Heat Shock Response (A) Mathematical equations.
(B) Lists of components and kinetic parameters (see also [10]).
(61 KB DOC)

## Abbreviations

CV: coefficient of variation
hsps: heat shock proteins
RNAP: RNA polymerase
