# Supplementary figures and images for: Module-Based Analysis of Robustness Tradeoffs in the Heat Shock Response System
Source: PLoS Comput Biol. 2006 Jul 28;2(7):e59. doi: 10.1371/journal.pcbi.0020059 (PMC1523291; doi:10.1371/journal.pcbi.0020059)

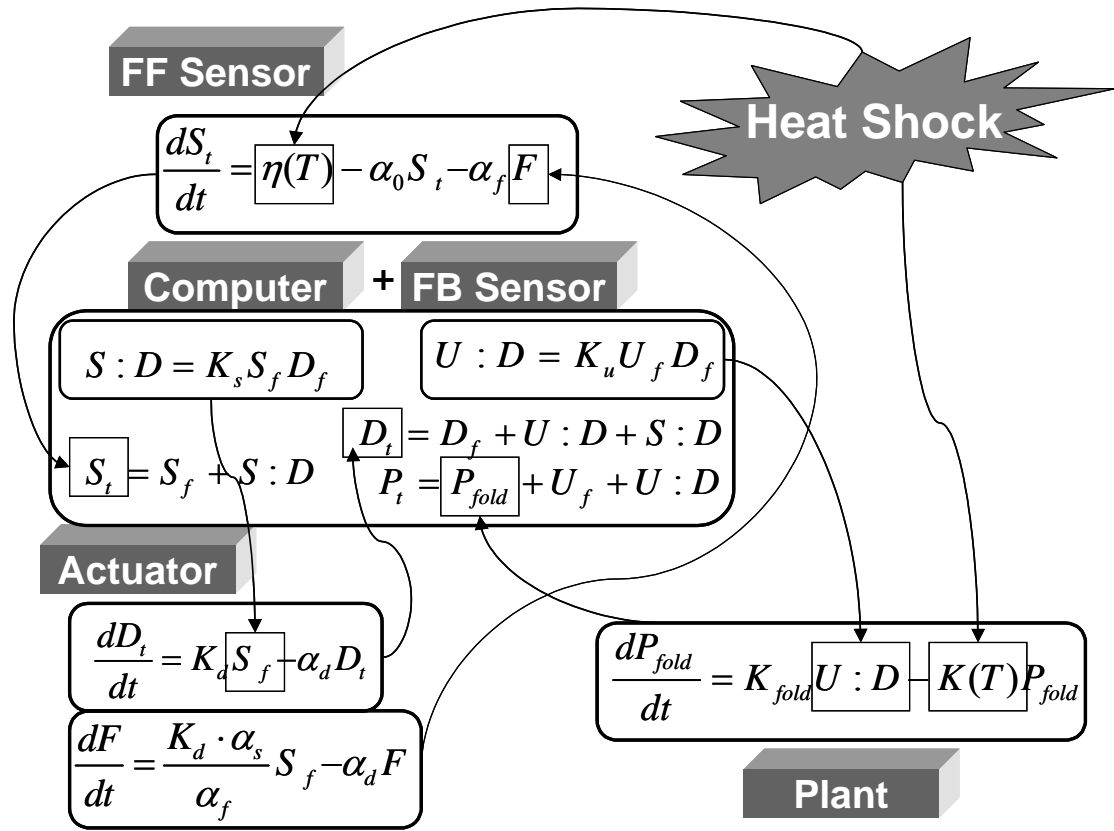

**Figure S1. Mathematical functional decomposition of the reduced order heat shock system.**

Supplement: Figure S1 — (75 KB PDF) [file pcbi.0020059.sg001.pdf]
