# Supplementary material for: Module-Based Analysis of Robustness Tradeoffs in the Heat Shock Response System
Source: PLoS Comput Biol. 2006 Jul 28;2(7):e59. doi: 10.1371/journal.pcbi.0020059 (PMC1523291; doi:10.1371/journal.pcbi.0020059)
Supplement: Figure S2 — (61 KB PDF) [file pcbi.0020059.sg002.pdf]

$$\frac{dS_t}{dt} = \eta(T) - \alpha_0 S_t - \alpha_f F \quad (1) \quad (4)$$

$$\frac{dD_t}{dt} = K_d S_f - \alpha_d D_t \quad (2)$$

$$\frac{dF}{dt} = \frac{K_d \cdot \alpha_s}{\alpha_f} S_f - \alpha_d F \quad (3) \qquad \left(F = \frac{\alpha_s}{\alpha_f} D_t\right)$$

$$\frac{dP_{fold}}{dt} = K_{fold} U : D - K(T) P_{fold}$$

Figure S2. Connection of flux modules to the differential equations describing the reduced qualitative model. (1) FF flux module, (2) SEQ-FB flux module, (3) DEG-FB flux module, and (4) σ^{32} amplifier flux module. The concentration of S_f integrates the effects of D, P_{fold}, and F, calculated from the algebraic equations.
