# Supplementary material for: Module-Based Analysis of Robustness Tradeoffs in the Heat Shock Response System
Source: PLoS Comput Biol. 2006 Jul 28;2(7):e59. doi: 10.1371/journal.pcbi.0020059 (PMC1523291; doi:10.1371/journal.pcbi.0020059)
Supplement: Figure S3 — (83 KB PDF) [file pcbi.0020059.sg003.pdf]

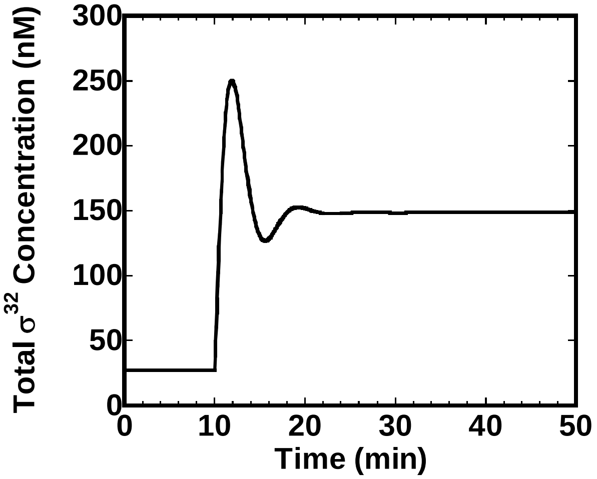

Figure S3. Time evolution of σ^{32} (S_t) for a reduced qualitative model of the heat shock response (**Table S1.**). Heat shock occurs at 10 min with an altered rate constant for denaturing proteins and translation for σ^{32}. *K(T)* is changed from 20 min^{-1} at low temperature to 60 min^{-1} at high temperature, while $\eta(T)$is changed from 7.5 x 10^{-8} M min^{-1} at low temperature to 4 x 75.0 at high temperature. The other parameters are α_d = 0.09 min^{-1}, α_0 = 0.09 min^{-1}, K_{fold} = 5000 min^{-1}, K_u = 1.0 x 10^{10} M^{-1}, P_t = 1.0 x 10^{-5} M. K_s = 1.0 x 10^{10} M^{-1}, K_d = 0.75 min^{-1}, α_s = 1.2 min^{-1}. The reduced order heat shock equations successfully reproduce the qualitative dynamic behavior of the heat shock response.
