# Supplementary material for: Module-Based Analysis of Robustness Tradeoffs in the Heat Shock Response System
Source: PLoS Comput Biol. 2006 Jul 28;2(7):e59. doi: 10.1371/journal.pcbi.0020059 (PMC1523291; doi:10.1371/journal.pcbi.0020059)
Supplement: Figure S4 — (65 KB PDF) [file pcbi.0020059.sg004.pdf]

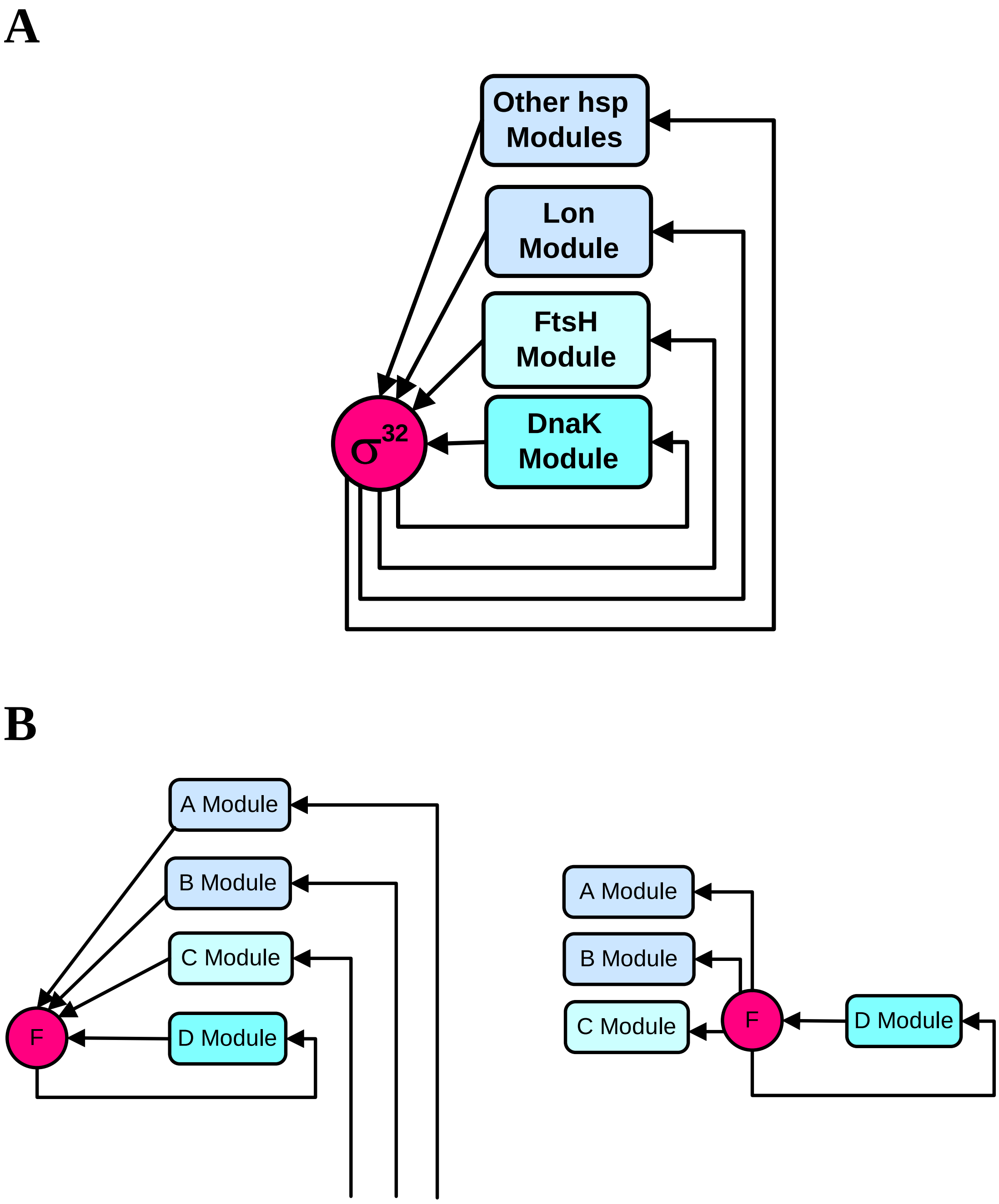

Figure S4. Definition of interconnected feedback loops. In the heat shock response system, modules are connected to σ^{32} using interconnected loops. (**A**) A typical example of interconnected feedback loops in the heat shock response. (**B**) A, B, and C modules do not form interconnected loops.
