# Supplementary material for: Module-Based Analysis of Robustness Tradeoffs in the Heat Shock Response System
Source: PLoS Comput Biol. 2006 Jul 28;2(7):e59. doi: 10.1371/journal.pcbi.0020059 (PMC1523291; doi:10.1371/journal.pcbi.0020059)

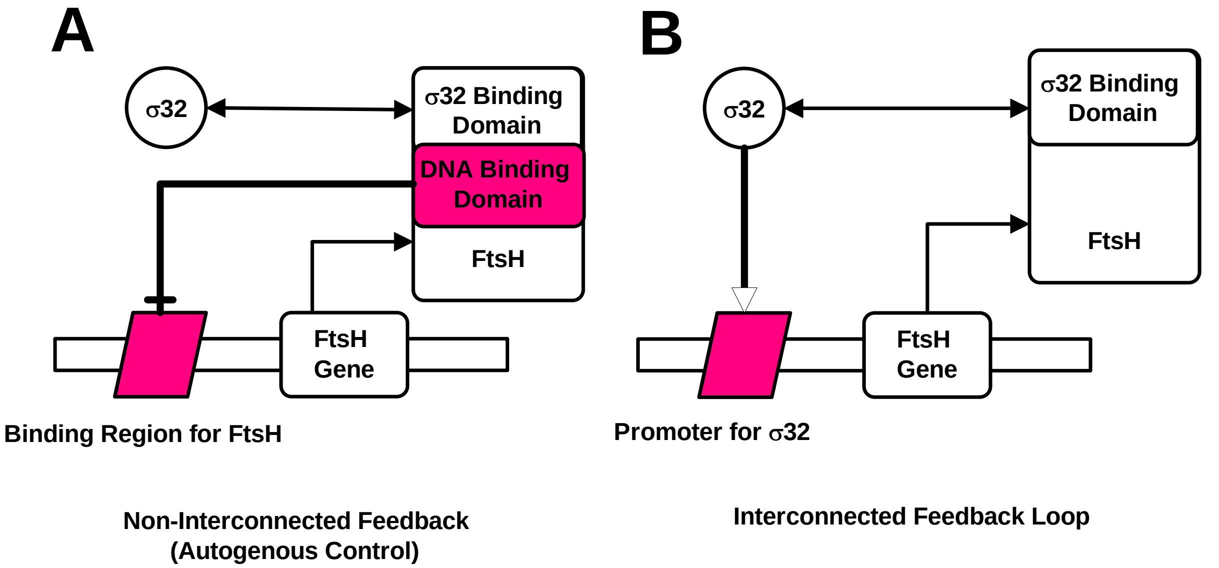

Figure S5. Protocol for interconnected feedback loops. Evolution of the DEG-FB loop is used as an example. For robust operation, a heat shock system lacking regulated degradation of σ^{32} by a member of its own regulon could evolve an independent controller that stabilizes the levels of FtsH tightly around its desired value (**A**). A more straightforward solution to the problem is to employ the protocol of an interconnected loop whereby the FtsH module is evolved just by fusing a promoter for binding of σ^{32} to the FtsH gene (**B**). In both cases, the red symbols must be added for a robust modular system.

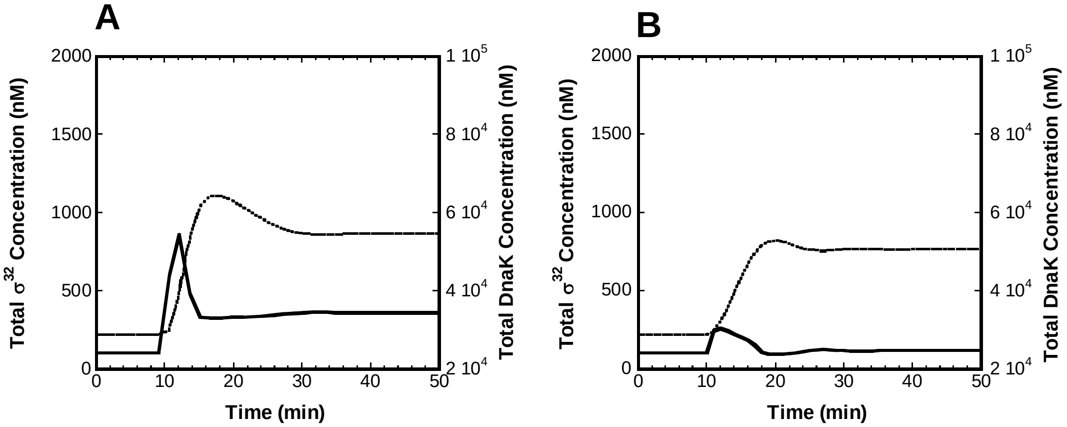

Supplement: Figure S5 — Evolution of the DEG-FB loop is used an example. (70 KB PDF) [file pcbi.0020059.sg005.pdf]
