# Supplementary material for: Module-Based Analysis of Robustness Tradeoffs in the Heat Shock Response System
Source: PLoS Comput Biol. 2006 Jul 28;2(7):e59. doi: 10.1371/journal.pcbi.0020059 (PMC1523291; doi:10.1371/journal.pcbi.0020059)
Supplement: Figure S6 — (70 KB PDF) [file pcbi.0020059.sg006.pdf]

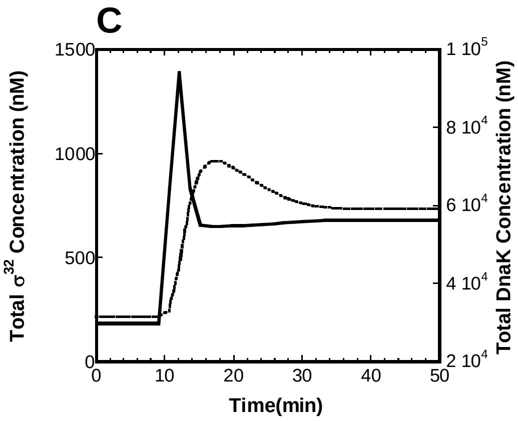

Figure S6. Time evolution of σ^{32} and DnaK for a detailed mechanistic model in the wild type and mutants. Heat shock occurs at 10 minutes and is implemented through an increase in the rate constant for protein denaturing. σ^{32}: ( ), DnaK: ( ). The parameters for the wild type are adjusted to provide a value of 0.99 for the yield at the steady state level at low temperature, and the efficiency is constrained to be more than 0.9 at high and low temperature. The values of the key parameters for each model are provided as follows. (**A**) Wild type (SEQ-FB + DEG-FB + FF system): *K[4]* = 3.2 x 10^6 M^{-1}, *km[1]* = 25.6 min^{-1}, *K[5]*= *K[6]* =1.0 x10^8 M^{-1}, *kx[1]* = *kx[2]* = 5 min^{-1}, *km[3]* = 20 min^{-1}, $\eta = 1$ (low temperature), $\eta = 4$ (at high temperature). (**B**) FF mutant (SEQ-FB + DEG-FB system): $\eta = 1$ (at high temperature). (**C**) FtsH knockout mutant (SEQ-FB + FF system): *km[3]* = 1 min^{-1}. (The transcription rate for FtsH is reduced by 95%, because the heat shock response has alternative proteases (HslVU, ClpAP, or Lon) whose function is similar to FtsH.)

The simulated time course trajectories for σ^{32} and chaperone reproduce the qualitative behavior of the wild-type heat shock response and mutants. At physiological temperatures (30-37 ^{o}C), there is a little σ^{32} present and hence a little transcription of the heat shock genes. When *E. coli* is exposed to high temperatures, σ^{32} first rapidly accumulates, allowing increased transcription of the heat shock genes, and then declines to a new steady state level characteristic of the new growth temperature (**A**). The FF-knockout mutant decreases the expression level of σ^{32}, which agrees with the experimental data (**B**). The FtsH-knockout mutant increases the σ^{32} level due to stabilization of σ^{32}. At high temperature, the σ^{32} concentration reaches the level of two-fold higher than that in the wild type, while the DnaK level does not increase so much due to feedback regulation by DnaK (**C**). These dynamic behaviors are consistent with the experimental data.

References:

1. Yura T, Nakahigashi K (1999) Curr Opin Microbiol 2: 153-158. 2. Morita MT, et al. (1999) Genes Dev 13: 655-665. 3. Tatsuta T, et al. (1998) Mol Microbiol 30: 583-593.
