# Supplementary material for: Module-Based Analysis of Robustness Tradeoffs in the Heat Shock Response System
Source: PLoS Comput Biol. 2006 Jul 28;2(7):e59. doi: 10.1371/journal.pcbi.0020059 (PMC1523291; doi:10.1371/journal.pcbi.0020059)
Supplement: Protocol S1 — (60 KB DOC) [file pcbi.0020059.sd001.doc]

### Protocol S1. Derivation of the sensitivity equation and sensitivity analysis.

Using the reduced order model (**Table S1**), we calculate analytically the concentration of DnaK and the sensitivity of the DnaK level (*D_t_*) to the change in the synthesis rate constant for DnaK (*K_d_*) at steady state. The expression for *D_t_* at steady state, obtained by setting the right hand side of the rate equations equal to zero, is given by:

$D_{t}=\frac{\eta(T)(K_{fold}+K(T))+\alpha_{0}K(T)P_{t}}{2(\alpha_{0}+\alpha_{s})(K_{fold}+K(T))}+\sqrt{\{\frac{\eta(T)(K_{fold}+K(T))+\alpha_{0}K(T)P_{t}}{2(\alpha_{0}+\alpha_{s})(K_{fold}+K(T))}\}^{2}+\frac{\eta(T)K_{d}}{\alpha_{d}K_{s}(\alpha_{0}+\alpha_{s})}}$.

The corresponding expression for the sensitivity at steady state, obtained by differentiating the equation above with respect to *K_d_*, is given by:

$\frac{\partial D_{t}}{\partial K_{d}}=\frac{\eta(T)(K_{fold}+K(T))}{\sqrt{K_{s}^{2}\alpha_{d}^{2}\{\eta(T)(K_{fold}+K(T))+\alpha_{0}K(T)P_{t}\}^{2}+4\eta(T)K_{d}K_{s}\alpha_{d}(\alpha_{0}+\alpha_{s})(K_{fold}+K(T))^{2}}}$.

These expressions use the following assumptions:

$U_{f}<<P_{t}-U:D$, $\frac{\eta(T)}{\alpha_{0}}+\frac{K(T)P_{t}}{K_{fold}+K(T)}>>\frac{1}{K_{s}}(1+\frac{\alpha_{s}K_{d}}{\alpha_{0}\alpha_{d}})$,$\frac{\alpha_{0}\alpha_{d}}{K_{d}}<<\alpha_{0}+\alpha_{s}$).

For the kinetic parameters that reproduce the dynamic behavior of the heat shock response (**Figure S3**), these assumptions are satisfied as shown below:

$\frac{U_{f}}{P_{t}-U:D}=0.001<<1$, $\frac{\frac{1}{K_{s}}(1+\frac{\alpha_{s}K_{d}}{\alpha_{0}\alpha_{d}})}{\frac{\eta(T)}{\alpha_{0}}+\frac{K(T)P_{t}}{K_{fold}+K(T)}}=0.003<<1$, $\frac{\frac{\alpha_{0}\alpha_{d}}{K_{d}}}{\alpha_{0}+\alpha_{s}}=0.008<<1$.

A careful scrutiny of these expressions reveals the following facts. First, the use of FF simultaneously increases the concentration of DnaK at steady state ($D_{t}$) and the sensitivity ($\frac{\partial D_{t}}{\partial K_{d}}$), confirming the intuition that FF is capable of providing sufficiently high yield but is very sensitive to parameter uncertainty. Second, an increase in the association constant between σ^32^ and DnaK (*K_s_*) simultaneously decreases the sensitivity ($\frac{\partial D_{t}}{\partial K_{d}}$) and the concentration of DnaK ($D_{t}$), which results in limiting the folded proteins. This analysis indicates that SEQ-FB cannot enhance the robustness of the system without reducing the concentration of DnaK. Therefore, this offers a further confirmation that the decreased DnaK is a tradeoff for the decreased sensitivity.
