# Supplementary material for: Module-Based Analysis of Robustness Tradeoffs in the Heat Shock Response System
Source: PLoS Comput Biol. 2006 Jul 28;2(7):e59. doi: 10.1371/journal.pcbi.0020059 (PMC1523291; doi:10.1371/journal.pcbi.0020059)
Supplement: Table S1 — (A) Mathematical equations. (B) Lists of components and kinetic parameters (see also [10]). (61 KB DOC) [file pcbi.0020059.st001.doc]

**Table S1.** Reduced Qualitative Model of the Heat Shock Response. (A) Mathematical equations. (B) Lists of components and kinetic parameters.

In order to generate an analytical tractable description that captures the essential dynamics of the heat shock response, we simplify the full order heat shock model into a compact set of equations. The simplified model is used to provide more insight into the intrinsic dynamical properties of the system and corroborate the results obtained numerically by simulating the full model. The simple model includes the dynamics of DnaK *(D_t_)* and the protease FtsH (*F)* in addition to their role in the regulation of σ^32^. The model also includes separation of fast and slow reactions and algebraic approximation for the dynamics of the fast reactions.

# (A)

| **Equations** |
| --- |
| $\frac{dS_{t}}{dt}=\eta(T)-\alpha_{0}S{}_{t}-\alpha_{f}F$ $\frac{dD_{t}}{dt}=K_{d}S{}_{f}-\alpha_{d}D_{t}$ $\frac{dF}{dt}=\frac{K_{d}\cdot\alpha_{s}}{\alpha_{f}}S{}_{f}-\alpha_{d}F$, ($F=\frac{\alpha_{s}}{\alpha_{f}}D_{t}$) $\frac{dP_{fold}}{dt}=K_{fold}U:D-K(T)P_{fold}$ $S:D=K_{s}S_{f}D_{f}$ $U:D=K_{u}U_{f}D_{f}$ $D_{t}=D_{f}+U:D+S:D$ $S_{t}=S_{f}+S:D$ $P_{t}=P_{fold}+U_{f}+U:D$ |

## (B)

| Components | | Kinetic parameters | |
| --- | --- | --- | --- |
| Symbol | Name | Symbol | Name |
| *S_t_* | total σ^32^ | *η(T)* | synthesis rate constant for σ^32^ (*T* : temperature) |
| *S_f_* | free σ^32^ | *α_o_* | degradation rate constant for σ^32^ |
| *S:D* | DnaK-bound σ^32^ | *α_f_* | FtsH-mediated degradation rate constant for σ^32^ |
| *D_t_* | total DnaK | *K_d_* | synthesis rate constant for DnaK |
| *D_f_* | free DnaK | *α_d_* | degradation rate constant for DnaK |
| *U:D* | DnaK-bound unfolded protein | *α_s_* | $\alpha_{s}=\alpha_{f}\frac{F}{D_{t}}$ |
| *F* | FtsH | *K_s_* | association constant between σ^32^ and DnaK |
| *P_t_* | total protein | *K_u_* | association constant between unfolded protein and DnaK |
| *P_fold_* | folded protein | *K_fold_* | refolding rate constant |
| *U_f_* | free unfolded protein | *K(T)* | denaturing rate constant (*T* : temperature) |
